# Chinese Veterinary Medicine B307 Promotes Cardiac Performance and Skeletal Muscle Contraction via Enhancing Intracellular Calcium Levels and Neural Electrical Activity in Animal and Cell Models

**DOI:** 10.1155/2020/9064824

**Published:** 2020-10-28

**Authors:** Chia-Ying Lien, Chen-Wen Lu, Chih-Hsiang Hsu, Tai-Yuan Chuang, Li-Yu Su, Wan-Jhen Wu, Yong-Sin Jheng, Ming-Chung Lee, Chung-Hsin Wu

**Affiliations:** ^1^Department of Athletics, National Taiwan University, Taipei City, Taiwan; ^2^School of Life Science, National Taiwan Normal University, Taipei City, Taiwan; ^3^Brion Research Institute of Taiwan, New Taipei City 23143, Taiwan

## Abstract

The study mainly investigated the effects of Chinese veterinary medicine B307 in cardiac and motor functions in animal models of pigeons and mice. Related cellular mechanisms were also studied in the neuroblastoma cell model of SH-SY5Y. Cardiac functions of pigeons and mice were examined by using moorFLPI Laser color Doppler imager and M-mode echocardiography, and motor functions were examined by using muscle electrical stimulation and force recording in the isolated breast muscle. Intracellular calcium levels and electrical activity of SH-SY5Y cells were examined by using Fura 2-AM fluorescence and MED64 system separately. Our results *in vivo* found that those pigeons under oral B307 treatment obviously enhanced subcutaneous microcirculation and contractile force and prolonged fatigue time in their breast muscles. Those mice under oral B307 treatment obviously elevated ejection fraction and cardiac output in their hearts. Our results *in vitro* showed that those SH-SY5Y cells under B307 treatment obviously increased intracellular calcium mobilization and electrical activities. These results revealed that improvement of cardiac and motor functions under B307 treatments may be caused by increasing electrical activities and intracellular calcium levels in neuromuscular cells and a similar mechanism may also occur in muscle cells. Thus, we suggested that B307 can be a functional Chinese veterinary medicine for flying pigeons.

## 1. Introduction

Chinese veterinary medicines (CVMs) as an alternative therapy for animals have become popular because of minimum side effects that are obviously different from Western veterinary medicines [1, 2]. For animal health considerations, the proportion of Chinese herbal medicines (CHMs) is usually determined based on scientific evidence and the experience of the prescriber [3]. As suggested in our previous studies, we have reported that CHM B307 can effectively enhance cardiac and motor functions for mice and rats [4–6]. CHM B307 with main herbal ingredients of ginseng (*Panax ginseng* Radix), schizandra (*Schisandra chinensis* Fructus), ophiopogon (*Ophiopogon spicata* Tuber), and Danshen (*Salviae miltiorrhizae* Radix) can be an appropriate CVM in protecting muscle against fatigue and strengthening the contraction of the heart muscle for animals [7–14]. Currently, CHM B307 has been widely used to enhance heart and muscle strength for the elderly especially. However, the possible cellular mechanisms of CHM B307 in improving cardiac and skeletal muscle contraction are still unclear and need to be clarified.

In Taiwan, pigeon racing is a form of entertainment for many people that is not just a sport but also a community. However, pigeons (*Columba livia domestica*) have been used by humans for meat supply, pet watch, sport racing, and experimental animals since 2500 BC [15]. In order to make racing pigeons stronger and healthier, they must undergo high training volume and intensity daily to improve their flight performance with better muscle endurance and speed. Additionally, supplementing food additives that can enhance physical strength such as CHM B307 should be an ideal CVM for flying pigeons. Under this consideration, we believe that the CVM B307 can be an ideal CVM for flying pigeons.

In this study, we provided concrete scientific evidence that B307 can improve cardiac and skeletal muscle contraction via enhancing intracellular calcium mobilization and firing activities in neuromuscular cells.

## 2. Materials and Methods

### 2.1. Chromatographic Fingerprint Analysis

CHM B307 is a herbal formula that was supplied by Sun-Ten Pharmaceutical Company (New Taipei City, Taiwan). The bioactive marker substances of B307 were analyzed by using chromatographic fingerprint analysis of high-performance liquid chromatography (HPLC, Thermo Fisher Scientific Inc., Waltham, MA, USA) that were graded with acetonitrile and methanol (Burdick & Jackson Korea, Seoul, Korea) and were qualitatively determined within 70 minutes under the selected HPLC condition. Ingredients of B307 were dissolved with purified water that was provided by the Milli-Q water purification system (Millipore, MA, USA).

### 2.2. Animal Models of Pigeons and ICR Mice

Six male pigeons (*Columba livia*) and twelve male ICR mice were used in this study. Pigeons were purchased from pigeons specialty store, while mice were purchased from BioLASCO Breeding Center (AAALAC International awarded, Yi-Lan, Taiwan). Animal experiments were permitted and supervised under the Institutional Animal Care and Use Committee of National Taiwan Normal University (Protocol number: NTNU Animal Experiments No. 104002). All animals were raised and cared according to the international guidelines for care and use of laboratory animals that were housed in the animal facility under specific pathogen-free conditions in a constant temperature environment at 22 ± 2°C with a 12 h light/dark cycle and had ad libitum access to water and food. Our animal experiments have been considered as the 3R spirit of “Replace”, “Reduce”, and “Refine” to optimize the experimental design.

### 2.3. Oral B307 Treatments in Animal Models

In this study, pigeons and ICR mice were orally treated with the CVM B307 extract (the pH value was closed to the 7.0) or their vehicle (dimethyl sulfoxide) in the drinking water at a dose of 30 mg/kg/day for 2 weeks. The reason why we used ICR mice is that ICR mice were derived from a noninbred strain in the Centre Anticancereux Romand laboratory in Switzerland. The strain has complete provenance data, is easy to buy, and has been widely used in general research, safety assessment tests, embryology research, and physiological research. As described in our previous experiments [4–6], the dosage of the B307 extract was much lower than the dosage of IC_50_. All protocols in the oral B307 treatments in animal models were executed according to the international guidelines for care and use of laboratory animals.

### 2.4. Subcutaneous Microcirculation Measurement

In this study, we scanned regionally dermal microvascular blood flows of pigeons with sham and B307 treatments without skin contact by using a Laser-Doppler imager (Moor Instruments, Axminister, UK). The method is as described in our previous experiment [16–22]; we positioned the camera of Laser-Doppler imager at 18 cm above the breast muscle of pigeons. Then, we recorded the subcutaneous blood flows of pigeons and then analyzed the dermal microvascular blood flows in arbitrary perfusion units (AU) with the data acquisition software (MoorFLPI measurement software, Version V3.0, Moor Instruments, Axminster, UK).

### 2.5. Cardiovascular Performance

Due to limitations of instrument function, we assessed the cardiovascular performance of mice instead of pigeons by color Doppler M-mode echocardiography. As described in our previous experiment [4, 5], after anesthesia with 2% isoflurane gas (Baxter Healthcare, New Providence, RI, USA), mice were placed on a heated working platform for monitoring ECG and respiration gating. Echocardiography was measured by using a prospect high-resolution imaging system (S-Sharp Corporation, Taiwan) with the probe providing the central frequency ranging from 20 to 40 MHz. By M-mode and color Doppler images, changes in heart rates, ejection fraction, and cardiac output were measured and compared between those mice with sham and oral B307 treatments. For all measures, three stable consecutive cardiac cycles were averaged for each mouse.

### 2.6. Isolated Whole Muscle Force Measurement

In this study, the muscle force of pigeons was mainly examined and compared with the whole skeletal muscle force of the isolated pectoralis major. Skeletal muscle force production can readily be measured in isolated whole breast muscle preparations of pigeons. The method is as described in the previous experiment [23]; we euthanized those pigeons by CO_2_ asphyxiation and confirm them by cervical dislocation; then we carefully dissected out an undamaged breast muscle that was entirely free of the skin. The isolated whole muscle was maintained in physiological salt solution at all times to ensure the muscle's viability and was affixed to stainless steel hooks for the purpose of attachment to a force transducer. Muscle contraction was triggered by electrically stimulating the breast muscle in the bath. Triggering pulses were generated by an electrical stimulator (Grass Instruments Model S88 Dual Output Square Pulse Stimulator; Warwick, RI, USA). The setup of force measurement was a commercial force transducer and A/D converter and computerized acquisition and analysis (World Precision Instruments, Sarasota, FL, USA).

### 2.7. SH-SY5Y Cell Culture and Growth

As described in our previous experiment [21], SH-SY5Y cells were cultured in a 1 : 1 mixture of Dulbecco's Modified Eagle's Medium and Ham's Nutrient Mixture F-12 with 100 *μ*g/mL streptomycin, 100 U/mL penicillin, and 10% fetal bovine serum (Thermo Fisher Scientific™). The SH-SY5Y cell culture medium was replaced every 48 hours, and cells were grown at 37°C in the presence of 5% CO_2_. The experiment of human neuroblastoma SH-SY5Y cell culture was approved under the approval of the Biological Experimental Safety Committee of the National Taiwan Normal University.

### 2.8. Measurement of Firing Activities

We measured and compared the firing activities between SH-SY5Y cells with sham and 2 *μ*g B307 treatments by using the MED 64 system (Alpha MED Scientific Inc. Osaka, Japan). The method is as described in the previous experiment [24], and in vitro neuroblastoma SH-SY5Y cell networks showed spontaneously bursting and spiking activity. The firing rates change between SH-SY5Y cells with sham and B307 treatments could be shown for each electrode that was very convenient to each channel of the MED 64 system. The standard deviation of each firing rate recording was used to estimate its spike threshold. A time interval of 300 ms was used to calculate the standard deviation of firing rates recording.

### 2.9. Measurement of Intracellular Calcium Levels

We measured and compared the calcium concentration of SH-SY5Y cells with sham and B307 treatments by using Fura-2, AM Calcium indicator (Thermo Fisher Scientific™), a green fluorescent calcium indicator. The method is as described in the previous experiment [24], SH-SY5Y cells (2 × 10^4^) were pretreated with vehicle or 2 *μ*g/mL B307 for 2 hours in an 8-well Nunc™ Lab-Tek™ culture chamber slide (Thermo Fisher Scientific™ cat#177445), and then the SH-SY5Y cells were washed twice in phosphate-buffered saline (PBS) and stained in the dark with 2 *μ*m Fura-2 AM for 40 minutes at 37°C in the presence of 5% CO_2_. Calcium fluorescence imaging Fura-2 AM was performed (*λ*Ex/Em = 340,380/512 nm) with an inverted microscope (Leica DM-IRB). We detected fluorescence intensity of SH-SY5Y cells by using ImageJ/FIJI software (NIH, http://fiji.sc/Fiji) that was compared as the cytosolic intracellular calcium concentration between SH-SY5Y cells with sham and B307 treatments.

### 2.10. Statistical Analysis

The data were obtained in at least three independent experiments. The values of the data were shown as the means ± standard error of the mean (SEM). Differences among groups with sham (or control) and B307 treatments were evaluated by one-way analysis of variance (ANOVA). The Student–Newman–Keuls multiple comparison post hoc test was performed if a significant F-value was observed. The *p* values of data were considered significant if they were at least <0.05.

## 3. Results

### 3.1. Chromatographic Fingerprint Analysis of the CVM B307

**Figure 1:**
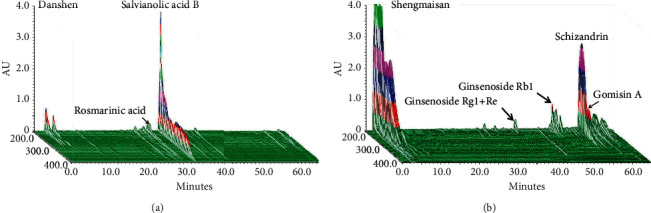
3D chromatographic fingerprint analysis of the CVM B307 by using high-performance liquid chromatography (HPLC). Bioactive marker substances for Danshen (*Salvia miltiorrhiza*) were Rosmarinic acid, and Salvianolic acid, B; Shengmaisan: Ginsenoside Rg1 ± Re, Ginsenoside Rb1, Schizandrin, and Gomisin A. Bioactive marker substances from ingredients of the B307 were qualitatively determined within 70 minutes under the selected HPLC condition. AU, arbitrary perfusion units.

Figure 1 shows the 3D chromatographic fingerprint analysis of the CVM B307 by using high-performance liquid chromatography. The main components of B307 are Danshen and Shengmaisan. The bioactive substances of Danshen (*Salvia miltiorrhiza*) are rosmarinic acid, and salvianolic acid B, while bioactive substances of Shengmaisan are Ginsenoside Rg1 ± Re, Ginsenoside Rb1, Schizandrin, and Gomisin A.

### 3.2. Effects of Oral B307 Treatment in Subcutaneous Microcirculation of the Pectoralis Major Muscle of Pigeons

**Figure 2:**
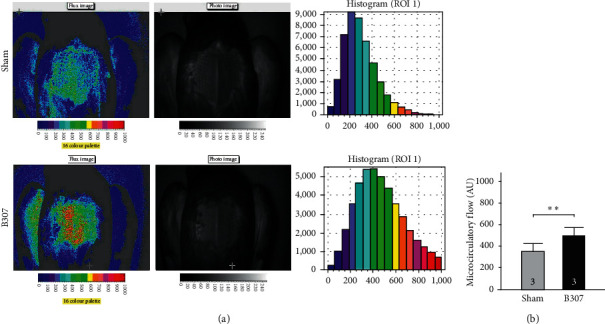
Comparison of subcutaneous microcirculation in pectoralis major muscle of pigeons under sham and oral B307 treatments. (a) Imaging of ventral microcirculatory flow in pectoralis major muscle of pigeons was enhanced under oral B307 treatment by moorFLPI Laser-Doppler imager. (b) Quantified subcutaneous blood flows of pectoralis major muscle of pigeons under oral B307 treatment were significantly greater than those of pigeons under sham treatment. The numbers of pigeons under sham and oral B307 treatments were 3 for each group. Values are mean ± SEM (^*∗∗*^*p* < 0.01, one-way ANOVA followed by a Student–Newman–Keuls multiple comparison post hoc test).

Figure 2(a) compares the imaging of subcutaneous microcirculatory flow in the pectoralis major muscle between pigeons under sham and oral B307 treatments. We found that the subcutaneous microcirculatory flow in the pectoralis major muscle of pigeons under oral B307 treatment was quite greater compared to that of pigeons under sham treatment. We quantified and compared subcutaneous blood flows in the pectoralis major muscle between pigeons under sham and oral B307 treatments in Figure 2(b). The statistical results show that those pigeons under oral B307 treatment significantly enhanced subcutaneous blood flow in the pectoralis major muscle than those pigeons under sham treatment (Figure 2(b)) (*p* < 0.01). Our results reveal that oral B307 treatment should have the promoting function of blood circulation for pigeons.

### 3.3. Effects of Oral B307 Treatment in Cardiac Performances of Mice

**Figure 3:**
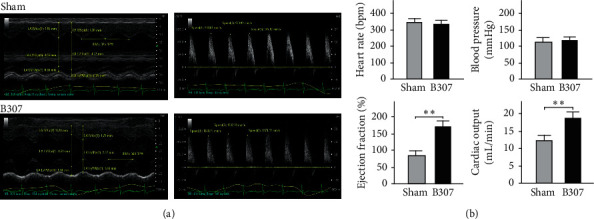
Comparison of cardiac performances of mice under sham and oral B307 treatments. (a) Echocardiographic pieces of evidence show systolic and diastolic cardiac performances in ICR mice under sham and oral B307 treatments. (b) Quantified heart rate and blood pressure of mice under oral B307 treatment were not significant than those of mice under sham treatment; while quantified ejection fraction and cardiac output of mice under sham treatment were significantly weaker than those of mice under oral B307 treatment. The numbers of mice under sham and oral B307 treatments were 6 for each group. Values are mean ± SEM (^*∗∗*^*p* < 0.01, one-way ANOVA followed by a Student–Newman–Keuls multiple comparison post hoc test).

Figure 3(a) compares the cardiac performances between mice under sham and oral B307 treatments. The reason why we assessed cardiovascular performance in mice instead of pigeons was due to limitations of the instrument function of echocardiography. We found that those mice under oral B307 treatment obviously enhanced systolic and diastolic cardiac performances than those mice under sham treatment. We quantified and compared heart rate, blood pressure, ejection fraction, and cardiac output between mice under sham and oral B307 treatments in Figure 3(b). The statistical results show that quantified heart rate and blood pressure of mice under oral B307 treatment were not more significant than those of mice under sham treatment (Figure 3(b)) (*p* > 0.05), while quantified ejection fraction and cardiac output of mice under oral B307 treatment were increased more significantly than those of mice under sham treatment (Figure 3(b)) (*p* < 0.01). Our results revealed that oral B307 treatment should have the promoting function of cardiac performances for mice.

### 3.4. Effects of Oral B307 Treatment in Muscle Force of Isolated Breast Muscle

**Figure 4:**
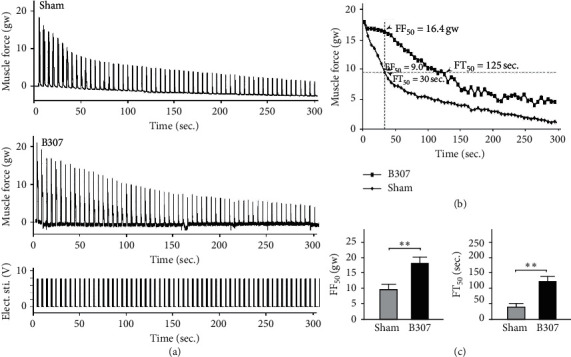
Comparison of contractile force and fatigue curve of the breast muscle in pigeons under sham and oral B307 treatments. (a) Contractile force of the isolated breast muscle in pigeons under sham treatment was obviously weaker over time than that of pigeons under oral B307 treatment. (b) Averaged fatigue curve of the breast muscle in pigeons under sham treatment was obviously steeper than that of pigeons under oral B307 treatment. Using a sham group of pigeons as a reference, the FF_50%_ and FT_50%_ are the contractile force and time at 50% of the fatigue curve. The values of the FF_50%_ and FT_50%_ were shown beside the curves. (c) Quantified FF_50%_ of the breast muscle in pigeons under sham treatment was significantly weaker than that of pigeons under oral B307 treatment (*p* < 0.01), while FT_50%_ of the breast muscle in pigeons under sham treatment was significantly shorter than that of pigeons under oral B307 treatment (*p* < 0.01). The numbers of pigeons under sham and oral B307 treatments were 3 for each group. Values are mean ± SEM (^*∗∗*^*p* < 0.01, one-way ANOVA followed by a Student–Newman–Keuls multiple comparison post hoc test).

Figure 4(a) compares the contractile force of isolated breast muscle in pigeons under sham and oral B307 treatments. We found that those pigeons under oral B307 treatment obviously increased the contractile force of isolated breast muscle more than those of pigeons under sham treatment. Our results reveal that oral B307 treatment should have the promoting function for the contractile force of the breast muscle in pigeons. We plotted the averaged fatigue curve of the breast muscle for pigeons under sham and oral B307 treatments in Figure 4(b). We found that the averaged fatigue curve of the breast muscle of pigeons under sham treatment was obviously steeper than that of pigeons under oral B307 treatment. In order to compare the contractile force and fatigue time of the breast muscle between pigeons under sham and oral B307 treatments, we used the 50% values in the fatigue curve of pigeons with sham treatment as a reference and defined the FF_50%_ and FT_50%_ as the contractile force and time at 50% of fatigue curve of the breast muscle. Our results showed that values of FF_50%_ and FT_50%_ were 9.0 gw and 30 sec for pigeons under sham treatment, while they were 16.4 gw and 125 sec for pigeons under oral B307 treatment (Figure 4(b)). We quantified and compared the values of FF_50%_ and FT_50%_ between pigeons under sham and oral B307 treatments in Figure 4(c). The statistical results show that quantified FF_50%_ of the breast muscle in pigeons under sham treatment was significantly less than that of pigeons under oral B307 treatment (Figure 4(c)) (*p* < 0.01), while quantified FT_50%_ of the breast muscle in pigeons under sham treatment was significantly shorter than that of pigeons under oral B307 treatment (Figure 4(c)) (*p* < 0.01). Our results revealed that oral B307 treatment should have the alleviating fatigue for the breast muscle of pigeons.

### 3.5. Effects of B307 Treatment in Electrical Activities of SH-SY5Y Cells

**Figure 5:**
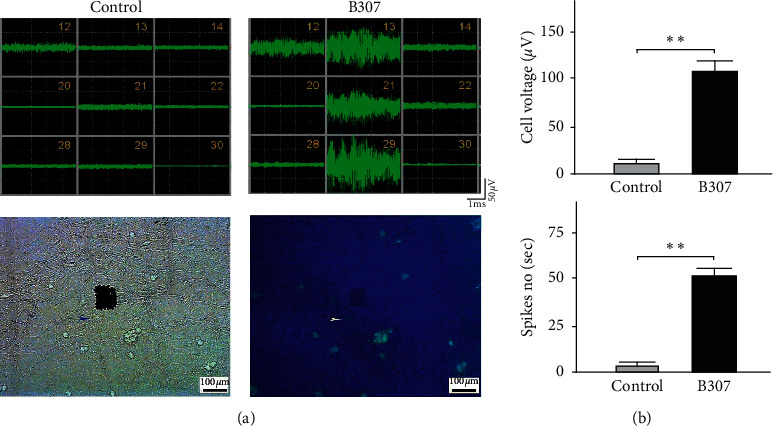
Comparison of electrical activities of SH-SY5Y cells before and during B307 treatments. (a) Electrical activities of SH-SY5Y cells before (Control) and during B307 (B307) treatments by using the MED64 system. The black square in the lower-left image indicated the recording probe, and the arrow indicated the recording SH-SY5Y cell. Scale bars: 100 *μ*m. (b) Quantified electrical voltage and (c) number of spikes per sec of SH-SY5Y cells were significantly enhanced during B307 treatment. The numbers of recording spikes were at least 100 for each SH-SY5Y cell. Values are mean ± SEM (^*∗∗*^*p* < 0.01, one-way ANOVA followed by a Student–Newman–Keuls multiple comparison post hoc test).

**Figure 6:**
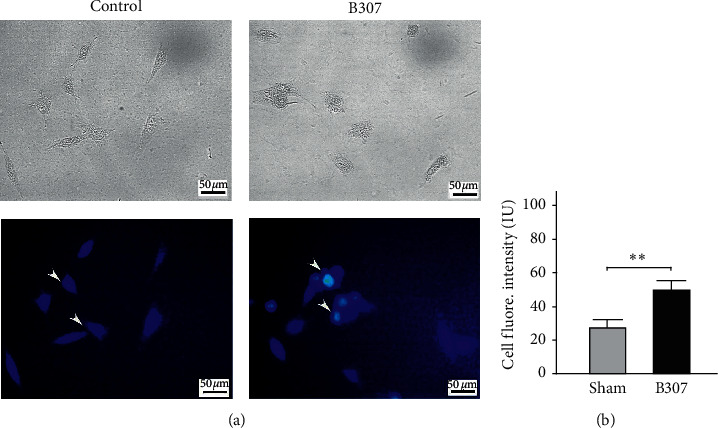
Comparison of calcium imaging of SH-SY5Y cells under sham and B307 treatments. (a) Living SHY5Y cells and their calcium imaging indicated that those cells obviously enhanced calcium imaging under B307 treatment. Scale bars: 50 *μ*m. (b) Quantified fluorescence intensity of calcium imaging in those cells under sham treatment was significantly weaker than that of cells under B307 treatment. The numbers of recording SHY5Y cells were 32. Values are mean ± SEM (^*∗∗*^*p* < 0.01, one-way ANOVA followed by a Student–Newman–Keuls multiple comparison post hoc test).

Figure 5(a) shows the electrical activities of SH-SY5Y cells before and during B307 treatments. The improvement of cardiac performance and skeletal muscle contraction may be through increasing firing activities in neuromuscular cells. Thus, we used SH-SY5Ycells as neural cells that may be more suitable for investigating the firing activities in neuromuscular cells than skeletal muscle cells. We found that those SH-SY5Y cells during B307 treatment obviously increased electrical activities than those of SH-SY5Y cells without B307 treatment. We quantified and compared the electrical voltage and spike number of SH-SY5Y cells before and during B307 treatments in Figures 5(b) and 5(c). The statistical results show that quantified electrical voltage and spike number of SH-SY5Y cells were significantly increased during B307 treatment (Figures 6(b) and 6(c)) (*p* < 0.01). Our results reveal that B307 treatment should have the promoting function of electrical activities for human neuroblastoma SH-SY5Y cells.

### 3.6. Effects of B307 Treatment in Intracellular Calcium Mobilization of SH-SY5Y Cells

Figure 6(a) shows living cells and their calcium imaging in SH-SY5Y cells under sham and B307 treatments by using Fluo-4 AM, a green fluorescent calcium indicator. We found that those SH-SY5Y cells under B307 treatment obviously enhanced the intracellular calcium levels than those SH-SY5Y cells under sham treatment. We quantified and compared the fluorescence intensity of calcium imaging between SH-SY5Y cells under sham and B307 treatments in Figure 6(b). The statistical results show that quantified intracellular calcium levels in SH-SY5Y cells under B307 treatment were significantly enhanced than those SH-SY5Y cells under sham treatment (Figure 6(b)) (*p* < 0.01). Our results reveal that oral B307 treatment should have the promoting function of intracellular calcium mobilization for human neuroblastoma SH-SY5Y cells.

## 4. Discussion

As an excellent racing pigeon, prolonged flight performance should be the necessary ability. When flying continuously, the flight muscle of a racing pigeon should be perfused sustainably with metabolites via the blood supply. Thus, better heart performance and stronger skeletal muscle contraction should be the key to determine the flight performance of racing pigeons. In this study, our results show that those pigeons under oral B307 treatment can improve subcutaneous microcirculation in pectoralis major muscles (Figure 2). Sustainable subcutaneous microcirculation via the blood supply in pectoralis major muscles may be caused by better cardiac performance. Our results provided evidence to confirm that those pigeons under oral B307 treatment can enhance both ejection fraction and cardiac output in the heart of mice (Figure 3). Under the influence of strengthening cardiac performance and increasing subcutaneous microcirculation, the pectoralis major muscle force should be stronger for racing pigeons. Our results confirm that those pigeons under oral B307 treatment can enhance the contractile force of pectoralis major muscles in pigeons (Figure 4). As suggested by fatigue curves of the isolated breast muscle in pigeons, we found that those pigeons under oral B307 treatment have the significantly greater contractile force and prolonged fatigue time in their pectoralis major muscles than those pigeons under sham treatment (Figures 4(b) and 4(c)). In other words, oral B307 treatment should improve contractile force and alleviate fatigue in the pectoralis major muscles of pigeons. Like most mammalian, avian species have a four-chambered heart. However, the skeletal and heart muscles of avian species need to be stronger and highly developed than normal mammals. This adaptive evolution enables avian species to more efficiently deliver nutrients and oxygen to the whole body to support the energy and metabolism required for flight. Thus, the effect of B307 on heart and muscle function should be more important than avian species. In addition, we have found that oral B307 treatment obviously elevated ejection fraction and cardiac output in the hearts of ICR mice. Like the evidence of oral B307 treatment enhancing skeletal muscle contractility, the echocardiographic pieces of evidence in Figure 3 showed that oral B307 treatment can enhance systolic and diastolic cardiac performances in ICR mice. Based on the above results, it is clear that oral B307 treatment has an enhancing effect on the contraction of both skeletal muscle and cardiac performances.

The chemical components of Chinese herbal medicines are not only complicated but also subject to many uncontrollable factors which indirectly affect their pharmacological effects. To this end, this experiment uses herbal extracts that have utilized chromatographic fingerprint analysis to ensure uniform quality and maintain and ensure the stability of its efficacy. The main components of B307 are Danshen and Shengmaisan (Figure 1). Danshen can alleviate heart disease and ameliorate the effects of atherosclerosis in humans [10, 11] and rodents [12]. Shengmaisan is a widely used formula in modern China for treating qi-deficiency syndromes and is comprised of three ingredients of ginseng, ophiopogon, and schizandra [25]. Ginseng contains a complex mixture of saponins, ginsenosides, and panaxosides, ophiopogon contains homoisoflavonoids with anti-inflammatory properties, and schizandra contains abundant amounts of phytoestrogen lignans with antioxidant activity [26]. As suggested in our previous studies, we have reported that B307 can effectively alleviate oxidative stress, inflammation, and damage in the myocardial tissue [4, 5]. In addition, our recent study also reported that B307 could be a protective and beneficial alternative treatment for thyroidectomy-induced cardiopulmonary exercise dysfunction [6]. To review the biological effects of the active substances in B307, previous studies have reported that ginseng has antioxidant [7], anti-inflammatory [8], and antifatigue [14] functions.

In vitro study, our results further confirmed that intracellular calcium levels and electrical activities of SH-SY5Y cells were obviously enhanced during B307 treatment (Figures 5 and 6). Ginseng is useful to enhance cardiac contractility in animals [27]. Recently, ginseng has been garnering increasing interest in its effects on the cardiovascular system and in the treatment of heart failure [28]. Ginsenosides, the effective component of ginseng, regulates cardiovascular function on cardiomyocyte contraction, which may be mediated in part through increased NO production [29]. Ginseng saponin, active ingredients of Panax ginseng, has also been reported to induce IP_3_-mediated Ca^2+^ release from endoplasmic reticulums for enhancing Ca^2+^-activated CI^−^ current [30]. We have observed that oral B307 treatment can promote intracellular calcium mobilization for human neuroblastoma SH-SY5Y cells. It is possible that ginseng, the active substance of B307, induces IP_3_-mediated Ca^2+^ release from endoplasmic reticulums. Our results further provided concrete scientific evidence that B307 could be an ideal functional CVM for flying pigeons because of the supplementary effects of cardiac performance and skeletal muscle contraction.

**Figure 7:**
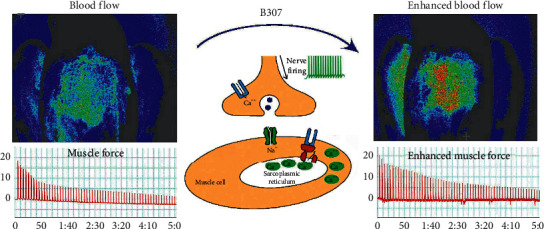
Schematic diagram illustrates that B307 could be an ideal CVM for flying pigeons because of improving cardiac performance and skeletal muscle contraction. Furthermore, the improvement of cardiac performance and skeletal muscle contraction may be through increasing firing activities and intracellular calcium levels in neuromuscular cells, and a similar mechanism may also occur in muscle cells.

Intracellular calcium mobilization is an important component of the signaling promoting striated (skeletal and cardiac) and smooth muscle contraction [31]. As suggested in the previous study [32], cardiac and skeletal muscle contraction requires increasing calcium levels of the cytosolic. In this study, we ever separately evaluated the pharmacological effects of effective contents of B307 in living SHY5Y cells. However, the effects of any component in electrical activities and calcium imaging were worse than the mixture. As for traditional Chinese medicine, we know that none of these contents have much therapeutic value. To use any of them alone may prove problematic. That is why we use combined herbal formulas to treat SHY5Y cells. The signaling pathways for muscle contraction should be generated by enhancing intracellular calcium levels from extracellular sources or released from intracellular stores. Rising cytosolic calcium may stimulate calcium-dependent signaling pathways and then regulate muscle contraction.  Figure 7 illustrates that B307 could be an ideal CVM for flying pigeons because of improving cardiac performance and skeletal muscle contraction. Furthermore, the improvement of cardiac performance and skeletal muscle contraction may be through enhancing intracellular calcium levels of muscle cells and increased firing activities of neuromuscular cells. It is possible that B307 treatments may enhance nerve firing and then increase acetylcholine (ACh) release from the endings of motor nerves. More end-plate potentials cause calcium channels to open and then cause the sarcoplasmic reticulum to release more calcium and finally to induce muscle contraction. As suggested in Figure 7, we consider that B307 treatment can modulate the calcium signaling pathways that generate cardiac and skeletal muscle contraction.

## 5. Conclusions

CHM B307 with main components of Danshen and Shengmaisan can modulate cardiac performance and skeletal muscle contraction in pigeons and mice. However, the improvement of cardiac and motor functions under B307 treatments may be caused by increasing electrical activities and enhancing intracellular calcium levels in neuromuscular cells, and a similar mechanism may also occur in muscle cells. Thus, we suggest that CHM B307 could be an ideal CVM for beneficial alternative treatment of racing pigeons.

## Data Availability

The data of chromatographic fingerprint of B307, immunohistochemistry, chemiluminescence, and biochemical analysis used to support the findings of this study are available from the corresponding author upon request.

## Abbreviations

CVM: MED64Chinese veterinary medicine
CHM: Chinese herbal 71 medicine
HPLC: High-performance liquid chromatography.
